# Correlation between weight-adjusted waist index and coronary heart disease: NHANES 1999–2020

**DOI:** 10.3389/fcvm.2024.1445802

**Published:** 2025-01-10

**Authors:** Yan Liu, Shougang Sun, Qi Zou, Ting Tao, Dian Li, Guodong Han, Zhiliang Wei

**Affiliations:** ^1^ The Second Clinical Medical School, Lanzhou University, Lanzhou, China; ^2^ Department of Cardiology, The Second Hospital of Lanzhou University, Lanzhou, China

**Keywords:** weight adjusted waist index, coronary heart disease, NHANES, obesity, epidemiology

## Abstract

**Background:**

The weight adjusted waist index (WWI) represents a novel indicator for assessing central obesity. The objective of this study is to investigate the association between WWI and coronary heart disease (CHD).

**Method:**

The data of 44,528 participants in total were gathered from NHANES database from 1999 to 2020. WWI is calculated as the waist circumference (WC, cm) divided by the square root of the body weight (kg), and CHD was determined based on participants’ self-reports. The association between WWI and CHD was examined using multiple logistic regression analysis, restrictive cubic spline (RCS), receiver operating characteristic (ROC) curve, mediation analysis, subgroup and interaction analyses.

**Result:**

This was a cross-sectional investigation. A total of 44,528 participants were included [50.23% male; mean WWI 10.89 (0.01) cm/√kg]. The multivariate logistic regression analysis revealed that in three models, one-standard-deviation increment in WWI was associated with an increased probability of CHD occurrence by 2.39 (2.22,2.57),1.47 (1.32,1.65), and 1.15 (1.00,1.32) times, respectively. Additionally, RCS analysis indicated a linear relationship between WWI and CHD. and the ROC analysis results showed that the discriminatory power of WWI for CHD was superior to that of body mass index (BMI) and WC. Glycated hemoglobin (HbA1c) partially mediated the relationship between WWI and CHD. Subgroup and interaction analyses confirmed that age, systolic blood pressure, and diabetes status had a significant impact on the association between WWI and CHD (*P* for interaction <0.05).

**Conclusion:**

The level of WWI has been demonstrated to be associated with an increased risk of CHD. Specifically, as WWI increases, the risk of CHD becomes higher. On this basis, it is hypothesized that WWI may potentially serve as an independent risk factor for CAD, thereby highlighting the substantial value of WWI in the identification and management of CHD.

## 1 Introduction

Coronary heart disease (CHD) is defined as a cardiovascular disorder arising from ischemia and hypoxia of myocardial tissue due to stenosis or occlusion of the coronary arteries. CHD, along with its associated complications such as myocardial infarction and heart failure, remains a major contributor to global morbidity and mortality rates (1, 2).The investigation revealed that the incidence rate of CHD had increased by 33% over the period from 1990 to 2019 (3),Additionally, in 2019, approximately 360,900 deaths in the American population were attributed to coronary heart disease (4).These data emphasize the detrimental effects of CHD and underscore the importance and urgency of proactive implementation of public health interventions for its prevention. Currently, a multitude of risk factors have been identified as being associated with CHD, encompassing smoking, hyperlipidemia, hypertension, diabetes, snoring, and obesity (1, 5, 6).

Obesity, which is recognized as one of the key risk factors for chronic diseases, poses a significant public health concern and demonstrates a close association with cardiovascular diseases. The primary etiologies underlying obesity relate to the increase in the amount and/or size of adipose tissue, as well as inappropriate adipose tissue distribution (7).According to the investigation, since 1980, the number of obese individuals has demonstrated a doubling trend. It is anticipated that by 2025, the prevalence of obesity among men will reach 18%, whereas among women, the prevalence is projected to exceed 21%. Furthermore, forecasts indicate that more than 6% of men and 9% of women will develop severe obesity (8, 9). The traditional metrics for assessing obesity, BMI and WC, are widely used. BMI, in particular, is the most commonly employed index due to its cost-effectiveness and ease of use. However, certain studies have identified a paradoxical relationship between BMI and the mortality rate associated with cardiovascular diseases (CVDs). Specifically, within certain populations, a higher BMI is not always directly associated with an increased risk of CVDs. In some instances, overweight or mildly obese individuals may even exhibit a lower risk or mortality rate of CVDs, a phenomenon referred to as the “obesity paradox” (10).Given the limitation of BMI in accurately distinguishing between fat mass and muscle mass, it is prudent to exercise caution when elucidating the relationship between obesity and CVDs, particularly focusing on CHD (10–12).WC is regarded as a preferred surrogate marker for quantifying visceral fat accumulation. However, it fails to account for the influence of height, thereby limiting its accuracy in assessing abdominal obesity across populations with varying stature (13).

In 2018, Park et al. introduced the WWI as an innovative metric to quantify the degree of obesity (14).This index integrates the advantages of WC while reducing the correlation with BMI, thereby enabling a more precise characterization of fat and muscle composition. Despite prior studies showing an association between WWI and CVDs mortality (15), the relationship between WWI and the incidence of CHD, as well as its comparative discriminatory ability for CHD vs. traditional indicators, remains unclear. Therefore, the aim of this study is to evaluate whether WWI can improve the detection rate of CHD in the general population.

## 2 Materials and methods

### 2.1 Description of the study

In this study, we utilized data from the NHANES database from 1999 to 2020. NHANES, administered by the National Center for Health Statistics (NCHS), aims to assess the health and nutritional status of adults and children in the United States. To ensure the representativeness of the sample, NHANES employed a multi-stage, stratified, clustered probability sampling design. Each participant underwent a series of assessments, and the collected data were subsequently categorized into five main sections: demographic information, dietary intake, physical examination results, laboratory test outcomes, and questionnaire responses. The NHANES protocol was approved by the Institutional Review Board of NCHS, and all participants provided written informed consent prior to their participation. Consequently, no further ethical review was required. Additionally, the data relevant to this study are publicly available on the NHANES official website (NHANES—National Health and Nutrition Examination Survey Homepage).

### 2.2 Study population

**Figure 1:**
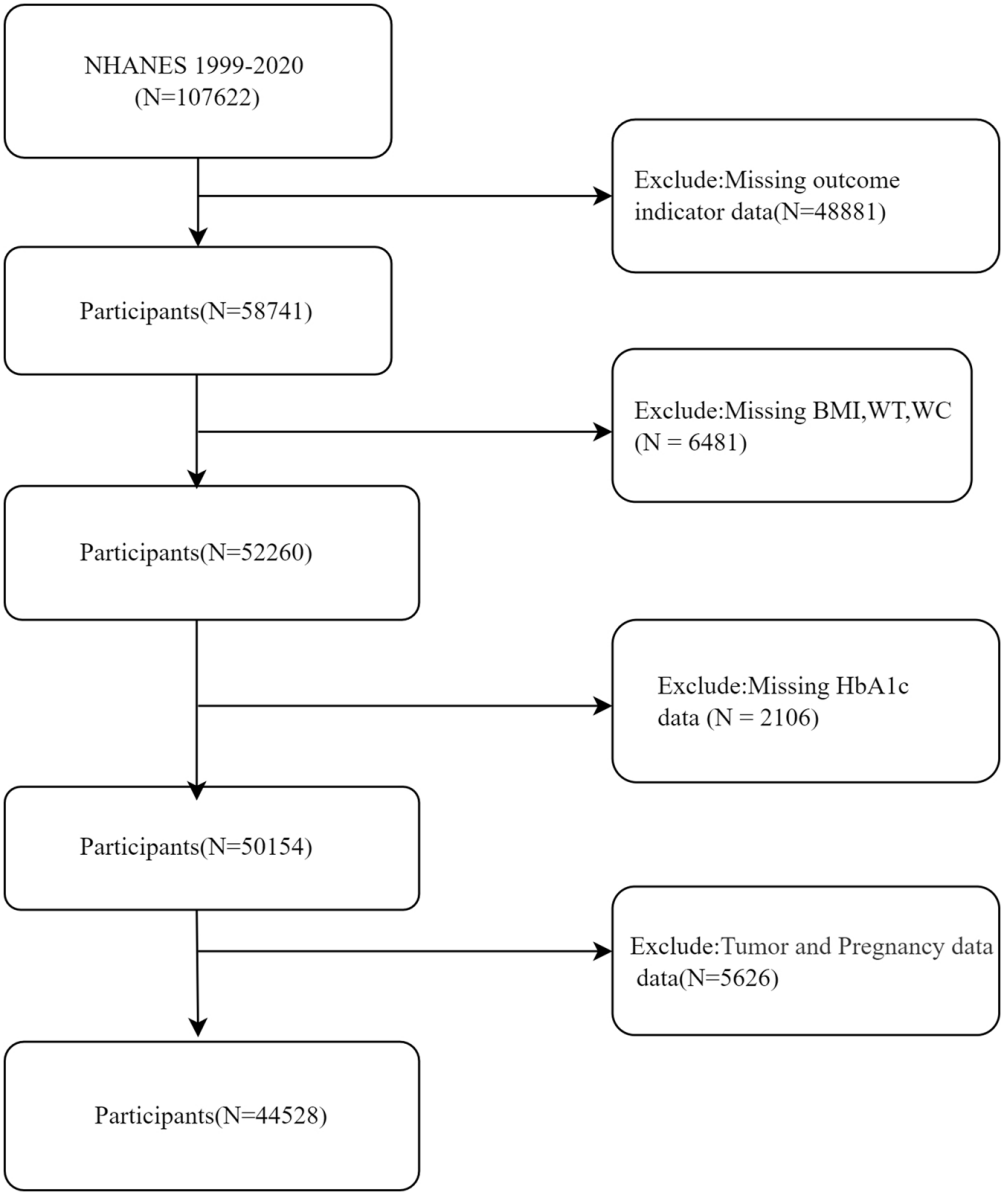
Flow chart of participant selection.

The present study included a comprehensive sample of 107,622 individuals sourced from the NHANES database, covering the period from 1999 to 2020. After rigorous exclusion criteria were applied, which involved removing individuals with missing or incomplete data on outcome measures (*n* = 48,881), physical morphological parameters (BMI, WC, weight) (*n* = 6,481), HbA1c (*n* = 2,106), as well as those with a history of tumors or current pregnancy (*n* = 5,626), a final cohort of 44,528 participants was included in the definitive analysis (Figure 1).

### 2.3 Assessment of WWI

In this study, the WWI (cm/√kg) was utilized as the exposure variable. It was calculated by dividing the WC (cm) by the square root of the body weight (kg). The WWI represents a novel metric for assessing obesity, with a particular emphasis on central obesity. A higher WWI ratio signifies a more severe degree of central obesity (14). The anthropometric parameters were collected by skilled health technicians within the Mobile Examination Center (MEC). During each physical measurement session, the technicians were assisted by recorders. Body weight was measured using a digital scale with a precision of 0.1 kg. Participants were positioned at the center of the scale, wearing standardized examination attire, with their hands placed alongside their bodies and gazing straight ahead. WC was assessed using a standard tape measure. A horizontal marking was made above the outermost edge of the right iliac crest, and the tape measure was positioned at the intersection of this line with the right mid-axillary line. The measurement was taken at the end of a normal exhalation and was recorded with a precision of 0.1 cm (16).

### 2.4 Assessment of CHD

The definition of CHD in our study was based on the response to the question in the questionnaire: “Has a doctor ever informed you that you have coronary heart disease?” Participants who responded “yes” were classified as belonging to the group with CHD. In our research, CHD was designated as the outcome variable.

### 2.5 Assessment of covariates

The other covariates included in this study were: age (≥60 years or <60 years), gender (male or female), race (non-Hispanic white, non-Hispanic black, other/multiracial, Mexican American, and other Hispanic American), alcohol consumption (no drinking, 1–5 times per month, 5–10 times per month, 10 + times per month), BMI (underweight: <18.5 kg/m^2^, normal weight: 18.5–24.9 kg/m^2^, overweight: 25.0–29.9 kg/m^2^, obese: ≥30.0 kg/m^2^), education level, smoking status, vigorous physical activity (yes or no), systolic blood pressure (SBP), diastolic blood pressure (DBP), cholesterol, creatinine, glucose, triglyceride, poverty index ratio (PIR), high-density lipoprotein cholesterol (HDL-C), uric acid, albumin level, HbA1c, heart failure status, and stroke status.

### 2.6 Statistical analysis

The statistical methodologies employed in this study adhered to the standards outlined on the NHANES official website. Given the multi-stage, stratified, and clustered probability sampling design, we applied appropriate weights to the analysis. In the baseline characteristics table, continuous variables were presented as mean (standard errors), while categorical variables were presented as weighted frequencies (weighted percentages). Descriptive statistical analysis of the WWI quartiles was conducted using the *t*-test or chi-square test. To examine the relationship between WWI and CHD, multiple logistic regression models were constructed. Prior to model building, variables with significant collinearity were excluded based on the correlation matrix and variance inflation factor (VIF). Three models were developed, guided by clinical expertise, AIC values, and likelihood ratio tests: Model 1, unadjusted for covariates; Model 2, adjusted for age, gender, race, education, and PIR; and Model 3, further adjusted for smoking, alcohol consumption, SBP, DBP, HDL-C, uric acid, triglycerides, albumin, physical activity, HbA1c, heart failure, stroke, BMI, and cholesterol. To assess the potential nonlinear relationship between WWI and CHD, RCS analysis was performed. Additionally, the ROC curve was utilized to compare the discriminatory abilities of BMI, WC, and WWI in determining CHD. Subsequently, mediation analysis was conducted to explore the role of HbA1c, as a mediator between WWI and CHD. Finally, subgroup analysis and interaction testing were employed to evaluate differences in the relationship between WWI and CHD across various subgroups, including age, gender, race, education, smoking status, alcohol consumption, SBP, DBP, and diabetes mellitus (DM) status. All statistical analyses were conducted using R version 4.3.3, with statistical significance set at a two-sided *P* < 0.05.

## 3 Result

### 3.1 Baseline data

The characteristics of the study population are presented in Table 1. A total of 44,528 participants were enrolled in this study [50.23% male; mean age 45.55(0.17)]. All covariates examined in this study demonstrated statistical significance in relation to the baseline characteristics across the WWI quartiles. The mean WWI value for all participants was 10.89(0.01) (cm/√kg). The specific quartile values of WWI were as follows: Q1:9.86 (0.01); Q2:10.60 (0.00); Q3:11.13 (0.00); and Q4:11.95 (0.01). Compared to individuals in the lowest WWI quartile, those in the highest quartile were more likely to be older, female, non-Hispanic white, and had a higher propensity for CHD and DM. Additionally, they exhibited higher levels of BMI, HbA1c, blood urea nitrogen, uric acid, triglycerides, blood glucose, SBP, and DBP. Concurrently, as the WWI increased, the PIR and HDL-C levels decreased.

**Table 1:** Baseline characteristics of the study participants.

	*n* ^a^	Overall *N* = 179,817,665^b^	Q1 *N* = 44,956,119^b^	Q2 *N* = 44,955,494^b^	Q3 *N* = 44,952,177^b^	Q4 *N* = 44,953,875^b^	*P*-value
Age (years)	44,528	45.55 (0.17)^c^	35.54 (0.20)	42.79 (0.22)	48.47 (0.20)	55.39 (0.24)	<0.001
WWI (cm/√kg)	44,528	10.89 (0.01)	9.86 (0.01)	10.60 (0.00)	11.13 (0.00)	11.95 (0.01)	<0.001
Gender (%)	44,528						<0.001
Female		90,860,401 (50.23%)^d^	19,578,207 (43.55%)	20,351,362 (45.27%)	22,082,941 (49.13%)	28,847,890 (64.17%)	
Male		88,957,264 (49.47%)	25,377,912 (56.45%)	24,604,131 (54.73%)	22,869,236 (50.88%)	16,105,985 (35.83%)	
Race (%)	44,528						<0.001
Non-Hispanic white		119,881,797 (66.67%)	30,304,752 (67.41%)	30,461,191 (67.76%)	29,261,199 (65.09%)	29,854,655 (66.41%)	
Non-Hispanic Black		20,084,725 (11.17%)	6,839,808 (15.21%)	4,554,201 (10.13%)	4,436,821 (9.87%)	4,253,895 (9.46%)	
Mexican American		15,703,032 (8.73%)	2,234,082 (4.97%)	3,830,351 (8.52%)	4,752,728 (10.57%)	4,885,871 (10.87%)	
Other/multiracial		13,039,585 (7.25%)	3,266,964 (7.27%)	3,362,834 (7.48%)	3,472,006 (7.72%)	2,937,782 (6.54%)	
Other Hispanic		11,108,525 (6.18%)	2,310,512 (5.14%)	2,746,917 (6.11%)	3,029,423 (6.74%)	3,021,673 (6.72%)	
PIR	44,528	2.990 (0.03)	3.149 (0.03)	3.163 (0.03)	2.987 (0.03)	2.661 (0.03)	<0.001
HBA1C (%)	44,528	5.562 (0.01)	5.243 (0.01)	5.416 (0.01)	5.622 (0.01)	5.965 (0.01)	<0.001
Nitrogen (mg/dl)	44,528	13.426 (0.05)	12.687 (0.07)	13.037 (0.07)	13.456 (0.07)	14.526 (0.08)	<0.001
DM (%)	44,528	32,520,856 (18.09%)	1,706,315 (3.80%)	4,400,235 (9.79%)	8,807,220 (19.59%)	17,607,085 (39.17%)	<0.001
Uric Acid (mg/dl)	44,528	5.396 (0.01)	5.077 (0.02)	5.359 (0.02)	5.503 (0.02)	5.645 (0.02)	<0.001
Triglycerides (mg/dl)	44,528	148.504 (1.08)	107.441 (1.39)	145.921 (1.64)	165.490 (1.79)	175.167 (1.89)	<0.001
Glucose (mg/dl)	44,528	105.371 (0.21)	96.743 (0.27)	101.496 (0.28)	107.043 (0.44)	116.203 (0.43)	<0.001
Albumin (g/dl)	44,528	4.287 (0.00)	4.413 (0.01)	4.334 (0.01)	4.255 (0.01)	4.147 (0.01)	<0.001
LDL-C (mg/dl)	44,528	113.235 (0.30)	105.795 (0.43)	115.302 (0.44)	117.473 (0.55)	114.371 (0.56)	<0.001
HDL-C (mg/dl)	44,528	52.967 (0.16)	57.163 (0.25)	53.240 (0.24)	51.140 (0.21)	50.324 (0.21)	<0.001
SBP (mmHg)	44,528	121.470 (0.15)	115.523 (0.19)	119.473 (0.22)	123.096 (0.22)	127.789 (0.25)	<0.001
DBP (mmHg)	44,528	71.416 (0.14)	69.536 (0.17)	72.279 (0.18)	72.505 (0.20)	71.374 (0.22)	<0.001
BMI (kg/m2)	44,528	28.749 (0.07)	24.432(0.06)	27.349(0.07)	29.855(0.07)	33.361(0.01)	<0.001

WWI, weight adjusted waist index; PIR, poverty-to-income ratio; CAD, coronary artery disease; HBA1C, glycated hemoglobin; DM, diabetes mellitus; LDL-C, low-density lipoprotein cholesterol; HDL-C, high-density lipoprotein cholesterol; SBP, systolic blood pressure; DBP, diastolic blood pressure; BMI, body mass index.

^a^ *n* (unweighted).

^b^ *N* (weighted).

^c^ For continuous: Mean (standard errors).

^d^ For categorical: *N* (weighted percentages).

### 3.2 Logistic analysis results

This study employed logistic regression analysis to assess the correlation between WWI and CHD, with the detailed results presented in Table 2. When WWI was treated as a continuous variable in the analysis, Model 1, which did not include any covariate adjustments, revealed that for each standard deviation increment in WWI, the odds of CHD occurrence significantly increased [OR (95%CI):2.39 (2.22, 2.57), *P* < 0.001]. After adjusting for factors such as age, gender, race, education, and PIR in Model 2, the increase in odds was attenuated to an OR of 1.47 (95%CI: 1.32, 1.65, *P* < 0.001). Upon further covariate adjustment based on Model 2, the odds increased to an OR of 1.15 (95% CI: 1.00, 1.32, *P* < 0.044) for each standard deviation increment in WWI. When WWI was analyzed using quartile grouping, a consistent trend was observed across all three models. Taking Model 1 as an example, with Q1 serving as the reference group, the odds of CHD occurrence in the Q2 group substantially increased [OR (95%CI): 3.39(2.41, 4.78), *P* < 0.001]; in the Q3 group, the odds further increased [OR (95%CI):6.07(4.18, 8.81), *P* < 0.001]; and in the Q4 group, the highest odds of CHD occurrence were observed [OR (95%CI):11.6(8.26, 16.4), *P* < 0.001]. After adjusting for corresponding covariates in Model 2 and Model 3, a similar trend of significantly increasing odds of CHD occurrence with increasing WWI quartiles was demonstrated, with all trend test *P*-values less than 0.05.

**Table 2:** Associations between WWI and risk of CAD in participants.

Exposure	Model1		Model2		Model3	
	OR^a^(95% CI^b^)	*P*-value	OR^a^(95% CI^b^)	*P*-value	OR^a^(95% CI^b^)	*P*-value
WWI (cm/√kg)	2.39 (2.22, 2.57)	<0.001	1.47 (1.32, 1.65)	<0.001	1.15 (1.00, 1.32)	0.044
WWI categories						
Q1	Ref		Ref		Ref	
Q2	3.39 (2.41, 4.78)	<0.001	1.81 (1.26, 2.58)	0.001	1.84 (1.29, 2.61)	<0.001
Q3	6.07 (4.18, 8.81)	<0.001	2.09 (1.41, 3.11)	<0.001	1.87 (1.25, 2.81)	0.003
Q4	11.6 (8.26, 16.4)	<0.001	2.83 (1.93, 4.15)	<0.001	1.98 (1.30, 3.00)	0.002
*P* for trend		<0.001		<0.001		0.003

Model 1 no covariates were adjusted. Model 2 adjusts for age, gender, race, education, PIR. Model 3 further adjusts for smoking, Alcohol status, SBP, DBP, HDL-C, uric acid, Triglycerides, Albumin, physical activity, Nitrogen, HBA1C, Heart Failure, Stroke, BMI, Cholesterol, DM.

^a^ OR, odds ratio.

^b^ CI, confidence interval.

### 3.3 RCS analysis result

**Figure 2:**
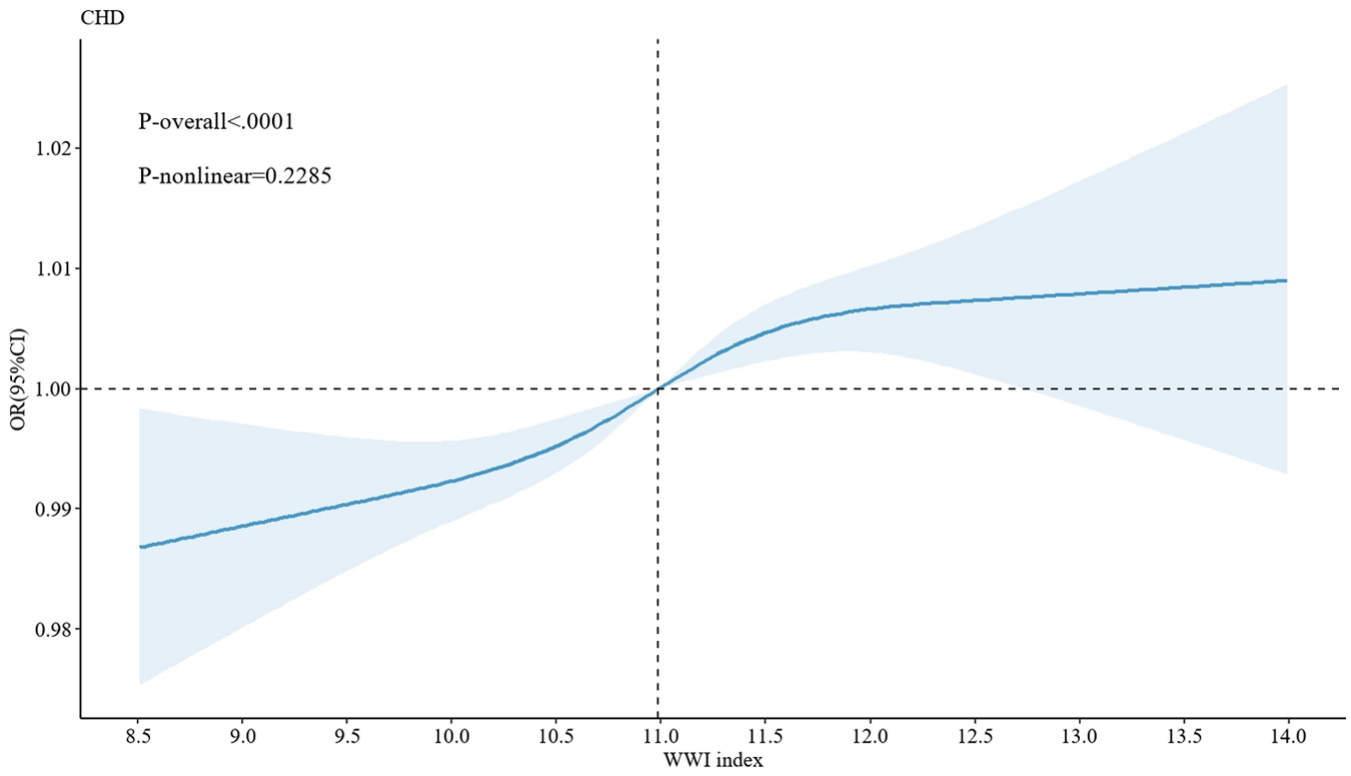
The nonlinear association between WWI and CHD.

Additionally, following the adjustment of all covariates in Model 3, an in-depth exploration was conducted using the generalized additive model (GAM) with smooth curve fitting capabilities. The results demonstrated a significant linear correlation between WWI and CHD (*P* for nonlinear = 0.2285, *P* < 0.05) (Figure 2). This finding further complemented and reinforced the results obtained from the previous logistic regression analysis, shedding light on the association between WWI and CHD from diverse perspectives.

### 3.4 ROC curve analysis and mediation analysis

**Figure 3:**
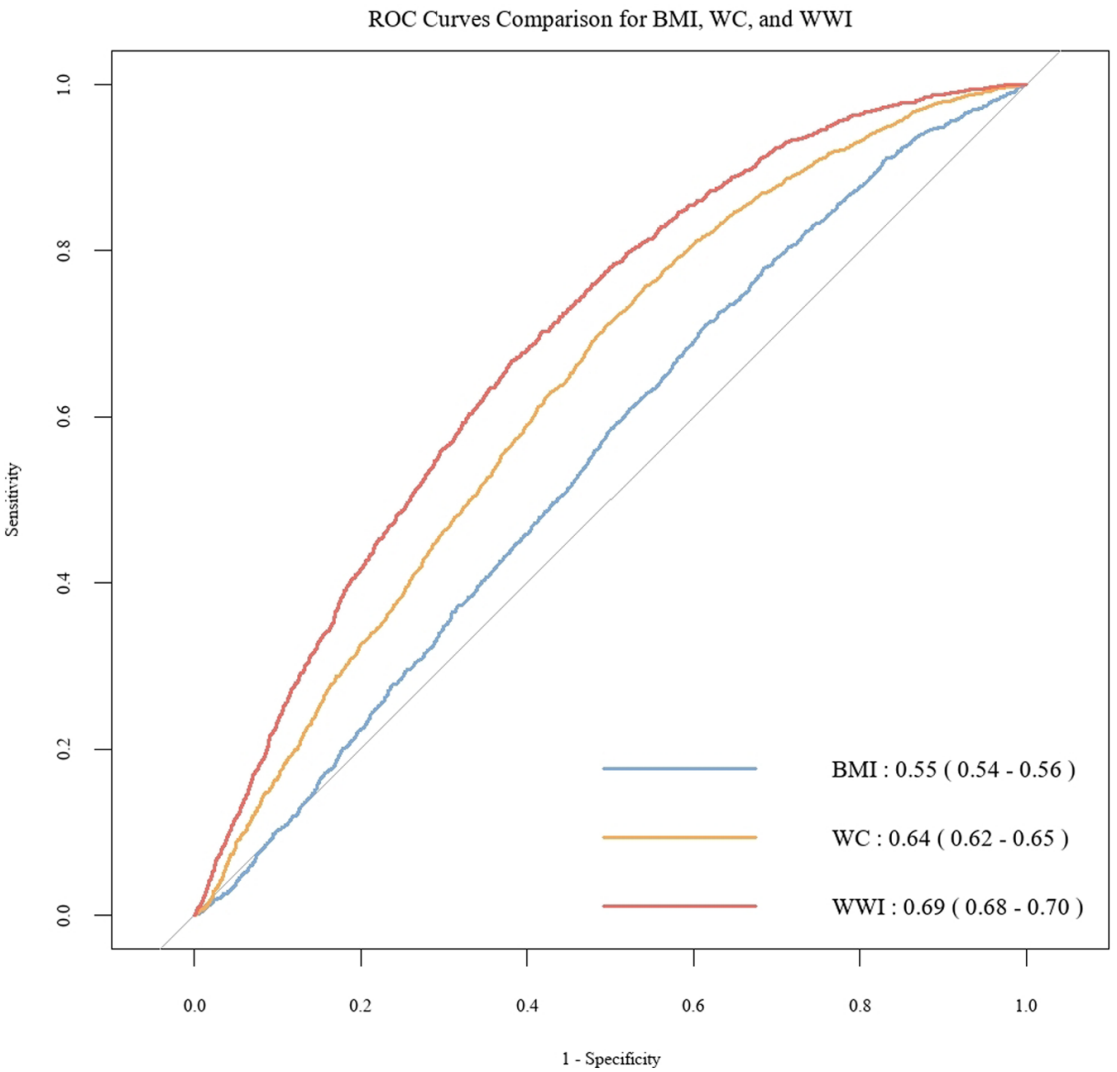
ROC curve for WWI, BMI, WC to discriminate the occurrence of CHD.

To further investigate the discriminatory abilities of WWI, BMI, and WC in relation to the risk of CHD, and to verify the superiority of WWI, we constructed ROC curves. The results demonstrated that WWI exhibited significantly stronger discriminatory power for CHD compared to BMI and WC [WWI: AUC(95%CI): 0.69(0.68, 0.70); BMI: AUC(95%CI): 0.55(0.54, 0.56); WC: AUC(95%CI): 0.64(0.62, 0.65)] (Figure 3). Additionally, the Table 3 illustrates the mediation analysis, The findings indicated that HbA1c partially mediated the association between WWI and CHD, with a mediation effect size of 16.4% (*P* < 0.001).

**Table 3:** Hba1c as a mediator in the associations between WWI and CHD.

Mediation effect	Estimate	95% CI lower	95% CI upper	*P*-value
Total effect	0.026	0.024	0.029	<0.001
Mediation effect	0.004	0.003	0.005	<0.001
Direct effect	0.022	0.020	0.024	<0.001
Proportion mediated	0.164	0.133	0.192	<0.001

### 3.5 Subgroup analyses and interactions

**Figure 4:**
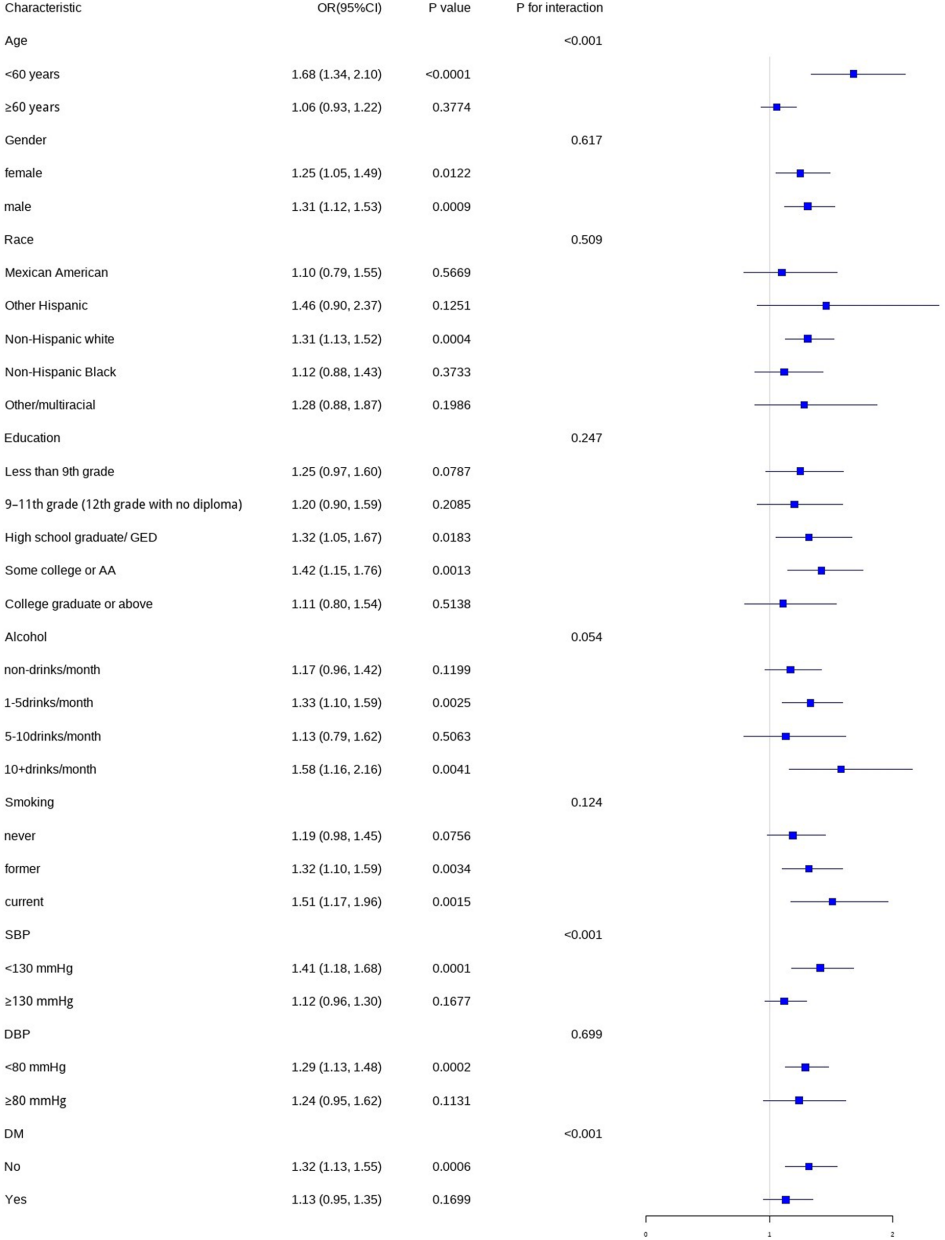
Subgroup analysis of the association between WWI and CHD.

In this study, we conducted subgroup analyses and interaction tests, stratified by age, gender, race, education level, smoking status, alcohol consumption, SBP, DBP, and DM status, to assess the robustness of the association between WWI and CHD and to detect any potential disparities (Figure 4). The results indicated that gender, race, education level, smoking status, alcohol consumption, and DBP did not significantly modify the relationship between WWI and CHD (*P* > 0.05). However, age, SBP, and DM status had a significant impact on the association between WWI and CHD (*P* < 0.05).

## 4 Discussion

In this cross-sectional study, which included 44,528 participants, we observed a linear and positive correlation between the WWI and the prevalence of CHD. This suggests that across the entire range of WWI values, individuals with higher WWI scores are more susceptible to developing CHD. Additionally, the results from the subgroup analysis and interaction tests indicated that age, SBP, and DM status had a specific impact on the stability of the relationship between WWI and CHD. The mediation analysis revealed that HbA1c partially mediated the association between WWI and CHD. When compared to BMI and WC, WWI demonstrated superior diagnostic utility for identifying an increased risk of CHD. Consequently, based on these findings, we hypothesize that an elevated WWI may serve as an independent risk factor for CHD, and its diagnostic accuracy for CHD surpasses that of BMI and WC, thereby highlighting the pivotal role of WWI in the prevention and management of CHD.

In addition to the present study, numerous prior investigations have explored the correlation between the WWI and various diseases. For instance, a study involving 12,447 participants demonstrated that a high WWI level (≥ 11.2 cm/√kg) was associated with an increase in all-cause mortality and cardiovascular mortality in southern China (17).In the field of cardiovascular diseases, Hu et al. also identified a significant positive correlation between the WWI and the risk of stroke. An increase in WWI indicates a higher risk of incident stroke (18).Concurrently, the study conducted by Zhang et al. also established a linear positive correlation between the WWI and the risk of heart failure. Specifically, for each standard deviation increment in WWI, the corresponding risk of heart failure increased by 19.5% (15).Furthermore, WWI may potentially serve as an intervention factor for the prevention and treatment of arteriosclerosis (19). in addition to its role in blood pressure management. Moreover, in a cohort study among patients with hypertensive obstructive sleep apnea (OSA), a J-shaped association was observed between WWI and CVDs. Notably, when WWI exceeded 11.5 cm/√kg, the risk of CVDs increased significantly (13).The correlations between WWI and a range of diseases, including bone mineral density (20), depression (21), chronic kidney disease (22), kidney stones (23), and fractures (24), have also been successively confirmed. Our study further confirmed the relationship between WWI and CHD, which aligns with previous research findings regarding the adverse effects of elevated WWI on CVDs.

Numerous prior studies have unequivocally demonstrated that obesity is a significant risk factor for CHD. Among these, BMI, serving as a conventional indicator for assessing obesity, has broad applications. Meyer et al. focused on children and adolescents aged 2–19 years and determined that BMI within this age range was positively correlated with the incidence of CHD in adulthood (25). Lamon-Fava et al. investigated the association between BMI and CHD separately in male and female groups, and their findings likewise demonstrated that an increase in BMI was adversely associated with the primary risk factors for CHD (26). In another study involving 15,828 patients with stable CHD, when BMI exceeded 25 kg/m^2^, cardiometabolic and inflammatory risk factors increased in a gradual manner concomitant with the increment in BMI (27). However, a study on the Korean population revealed a U-shaped association between BMI and all-cause mortality, CVDs mortality, and cancer mortality. The phenomenon whereby the mortality risk of moderate obesity is lower than that of normal weight, underweight, or overweight individuals is termed the “obesity paradox” (28). This phenomenon is partly attributed to methodological biases or the confounding effects of other factors, and it also highlights the limitations of BMI in differentiating between fat mass and lean body mass (10, 12). On the basis of traditional indicators, researchers have been persistently exploring new obesity assessment indicators, such as WC, Although WC is also a commonly used measure of obesity, due to its high correlation with BMI, it has not emerged as an outstanding obesity assessment indicator independent of BMI (29).

A pivotal finding of this study is that HbA1c partially mediates the association between WWI and CHD. HbA1c serves as an indicator of blood glucose levels over the preceding 2–3 months and is a crucial diagnostic criterion for DM. Studies have shown a positive correlation between HbA1c levels and the incidence and mortality rates of cardiovascular diseases (30). Therefore, maintaining optimal HbA1c levels is beneficial for the prevention of CHD and the improvement of prognosis in CHD patients (31).

As a novel indicator for assessing central obesity, the WWI demonstrates significant advantages over the conventional BMI and WC in determining the risk of CHD. By combining WC with the square root of body weight, the WWI not only retains the strengths of WC in evaluating fat distribution but also reduces its correlation with BMI, thereby providing a more comprehensive reflection of an individual's muscle mass and fat mass. This advantage persists even among individuals with varying BMI levels (32). Furthermore, WWI mitigates the influence of the obesity paradox, Consequently, it emerges as a more comprehensive and precise tool for CHD risk assessment, possessing substantial clinical application value (18). A vast array of studies has established a notable correlation between central obesity and the risk and mortality of CHD. For example, Coutinho et al. conducted a comprehensive analysis of 2,188 studies and found that central obesity was directly linked to CHD mortality (33).Similarly, a study following 130,473 participants aged 60–69 in the UK Biobank further confirmed this finding (34). As research progresses, an ever-growing body of evidence suggests that WWI is associated with a range of diseases, including hypertension, arteriosclerosis, abnormal uric acid levels, heart failure, and depression (15, 19, 35–37).Our study also confirms that WWI may serve as an independent risk factor for CHD. Compared to the traditional BMI anthropometric parameter, WWI more accurately reflects the distribution of body fat and proposes a novel strategy for the prevention and treatment of coronary heart disease (16).

The WWI exhibits a positive correlation with abdominal fat mass and a negative correlation with abdominal muscle mass, enabling it to reflect the ratio of fat to muscle mass rather than an absolute fat mass alone (38).Consequently, compared to BMI and WC, WWI is more adept at accurately capturing “true obesity,” specifically the form of obesity intimately associated with metabolic disturbances. The mechanisms underlying the increased risk of CHD associated with elevated WWI values can be elucidated from two perspectives: Firstly, abdominal fat cells, particularly visceral fat cells, have a greater propensity to directly release free fatty acids (FFAs) into the portal vein compared to other anatomical regions. This results in prolonged exposure of the liver to a high FFA milieu, stimulating the synthesis of very low-density lipoprotein (VLDL), which subsequently elevates plasma triglyceride and cholesterol levels, leading to dyslipidemia. Additionally, the high FFA environment may induce insulin resistance, further augmenting blood pressure and insulin levels (hypertension and hyperinsulinemia), and elevating blood glucose, thereby increasing HbA1c levels. The chronic hyperglycemic state indicated by high HbA1c levels intensifies the inflammatory response, collectively promoting the development of atherosclerosis (33, 39, 40). Secondly, the augmentation in central obesity denoted by WWI may reflect perturbations in fat metabolism. This, in turn, prompts the body to release more reactive oxygen species (ROS). These ROS interact with nitric oxide (NO) to produce cytotoxic peroxynitrite, damaging vascular endothelial cells. Furthermore, the increase in ROS accelerates the oxidation of low-density lipoprotein (LDL), making it more susceptible to phagocytosis by macrophages and the formation of foam cells, thereby exacerbating vascular endothelial damage and facilitating atherosclerosis (41–43).In summary, WWI, as an index that concurrently reflects the ratio of fat to muscle mass, provides a novel perspective for understanding and assessing obesity and its association with metabolic disorders, such as CHD. With further research and validation, WWI is anticipated to become a crucial tool in the management of obesity and the prevention of CHD.

One of the prominent strengths of this study is its large-sample size, which is based on the NHANES database. The stratified, multi-stage, and clustered probability sampling paradigm employed by this database ensures the representativeness and reliability of the data. During the data analysis process, we utilized a correlation matrix and variance inflation factor to preempt multicollinearity and further compared the discriminative abilities of WWI, BMI, and WC for CHD using the ROC curve. Additionally, our study determined that HbA1c partially mediates the correlation between WWI and CHD. Furthermore, we conducted subgroup analyses and interaction assessments to thoroughly investigate the disparities in the correlation between WWI and CHD across diverse populations. Despite these strengths, our study also has certain limitations. Firstly, since the data is primarily sourced from the American population, caution should be exercised when generalizing the results to populations in other countries and regions. Secondly, due to the cross-sectional nature of the NHANES database, our study is unable to directly infer a causal relationship between WWI and coronary heart disease. Additionally, the outcome variable used in this study was derived from a questionnaire survey, which may introduce recall bias or misclassification. Finally, although we made efforts to adjust for potential covariates during data analysis, there may still be other unaccounted factors that could impact the results. Therefore, in future studies, we will further explore these issues and work to resolve these limitations.

## 5 Conclusion

In conclusion, it clearly demonstrates a positive correlation between the level of WWI and the risk of CHD. Specifically, an elevation in WWI is accompanied by a corresponding increase in the risk of CHD. Notably, compared to traditional indices, WWI exhibits superior diagnostic potential for CHD. Furthermore, the study also established that HbA1c partially mediates the relationship between WWI and CHD. These findings strongly suggest that WWI may be an independent risk factor for CHD, thereby opening up novel potential strategic pathways for the prevention and treatment of coronary heart disease. Given that WWI possesses notable advantages such as ease of calculation, convenient acquisition, and high cost-effectiveness, its widespread application within various medical and health institutions is highly feasible. Especially in regions with relatively scarce medical and economic resources, the implementation of WWI may yield substantial social and economic benefits. Consequently, further in-depth exploration of the relationship between WWI and CHD is of great significance. Looking ahead, research could be considered to advance in the following areas: Firstly, conduct large-scale longitudinal studies to elucidate the causal link between WWI and CHD, thereby providing more compelling evidence for disease prevention and control. Secondly, perform studies among different ethnic and regional populations to validate the universality of WWI in predicting CHD and make the research findings more widely applicable. Thirdly, deeply investigate the underlying mechanism through which WWI influences CHD, dissect the changes at the cellular and molecular levels, and concurrently explore the impact of lifestyle interventions on the relationship between WWI and CHD. Through these efforts, it is anticipated that the value of WWI as a CHD predictor will be more comprehensively and profoundly affirmed, thereby establishing a solid theoretical and practical foundation for the precise prevention and treatment of CHD.

## Data Availability

The datasets presented in this study can be found in online repositories. The names of the repository/repositories and accession number(s) can be found in the article/Supplementary Material.
